# Molecular epidemiology of tuberculosis in the Somali region, eastern Ethiopia

**DOI:** 10.3389/fmed.2022.960590

**Published:** 2022-10-14

**Authors:** Getnet Worku, Balako Gumi, Binyam Mohammedbirhan, Musse Girma, Henok Sileshi, Michael Hailu, Amanuel Wondimu, Wondimu Ashagre, Rea Tschopp, Lauren Carruth, Gobena Ameni

**Affiliations:** ^1^Department of Medical Laboratory Science, College of Medicine and Health Sciences, Jigjiga University, Jigjiga, Ethiopia; ^2^Aklilu Lemma Institute of Pathobiology, Addis Ababa University, Addis Ababa, Ethiopia; ^3^Department of Pathology, College of Medicine and Health Sciences, Jigjiga University, Jigjiga, Ethiopia; ^4^National Tuberculosis Reference Laboratory, Ethiopian Public Health Institute, Addis Ababa, Ethiopia; ^5^One-Health Unit, Armauer Hansen Research Institute, Addis Ababa, Ethiopia; ^6^Department of Epidemiology and Public Health, Swiss Tropical and Public Health Institute, Basel, Switzerland; ^7^School of International Studies, American University, Washington, DC, United States; ^8^Department of Veterinary Medicine, College of Agriculture and Veterinary Medicine, United Arab Emirates University, Al Ain, United Arab Emirates

**Keywords:** molecular epidemiology, tuberculosis, spoligotyping, eastern Ethiopia, *M. tuberculosis* complex

## Abstract

**Background:**

Tuberculosis (TB) is one of the leading causes of morbidity and mortality in low-income countries like Ethiopia. However, because of the limited laboratory infrastructure there is a shortage of comprehensive data on the genotypes of clinical isolates of *Mycobacterium tuberculosis* (*M. tuberculosis*) complex (MTBC) in peripheral regions of Ethiopia. The objective of this study was to characterize MTBC isolates in the Somali region of eastern Ethiopia.

**Methods:**

A cross-sectional study was conducted in three health institutions between October 2018 and December 2019 in the capital of Somali region. A total of 323 MTBC isolates (249 from pulmonary TB and 74 from extrapulmonary TB) were analyzed using regions of difference 9 (RD 9)-based polymerase chain reaction (PCR) and spoligotyping.

**Results:**

Of the 323 MTBC isolates, 99.7% (95% CI: 99.1–100%) were *M. tuberculosis* while the remaining one isolate was *M. bovis* based on RD 9-based PCR. Spoligotyping identified 71 spoligotype patterns; 61 shared types and 10 orphans. A majority of the isolates were grouped in shared types while the remaining grouped in orphans. The *M. tuberculosis* lineages identified in this study were lineage 1, 2, 3, 4, and 7 with the percentages of 7.4, 2.2, 28.2, 60.4, and 0.6%, respectively. Most (87.9%) of the isolates were classified in clustered spoligotypes while the remaining 12.1% isolates were singletons. The predominant clustered spoligotypes identified were SIT 149, SIT 21, SIT 26, SIT 53, and SIT 52, each consisting of 17.6, 13.3, 8.4, 7.4, and 5%, respectively. Lineage 3 and lineage 4, as well as the age group (15–24), were associated significantly with clustering.

**Conclusion:**

The MTBC isolated from TB patients in Somali region were highly diverse, with considerable spoligotype clustering which suggests active TB transmission. In addition, the Beijing spoligotype was isolated in relatively higher frequency than the frequencies of its isolation from the other regions of Ethiopia warranting the attention of the TB Control Program of the Somali region.

## Introduction

Tuberculosis (TB) is a bacterial infection caused by the *M. tuberculosis* complex (MTBC) and affects any part of the human body, although it most commonly affects the lungs. It is transmitted through inhalation and is a major cause of morbidity and one of the top causes of mortality worldwide. Until the coronavirus (COVID-19) pandemic, TB was the leading cause of mortality caused by a single infectious agent, surpassing HIV/AIDS. According to the latest report, WHO estimated 9.9 million cases of TB and 1.5 million deaths in 2020, and an additional 214,000 deaths resulting from TB disease among people living with HIV (1).

Genotyping approaches for *M. tuberculosis* have proved to be valuable in acquiring a better understanding of TB epidemiology, which is important for effective TB control strategies (2), such as, detecting distinct strains that spread in epidemics (3), identifying recurring TB attributable to external reinfection or relapse (4), and detecting laboratory cross-contamination (5). Furthermore, the establishment of a phylogenetic framework for *M. tuberculosis* due to variances in their genetic makeup has allowed researchers to investigate the public health consequences of different genotypes of MTBC such as transmission rate, drug resistance development, immunological responses, and disease severity (6).

In Ethiopia TB remains one of the leading public health concerns claiming the lives of thousands of Ethiopians every year. Ethiopia is among 30 countries with a high TB burden and 30 countries with a high TB/HIV burden throughout the world and has recently been removed from the WHO's list of 30 countries with a high multidrug resistant (MDR) TB burden (1). Previous molecular epidemiology studies in different regions of Ethiopia revealed that lineage 4 and lineage 3 were predominant, whereas lineage 1 and lineage 2 were the least common. Lineage 7 (Ethiopian) appeared to be geographically restricted to northern Ethiopia. The most prevalent clades/families found in the country were T, CAS, H, Manu, and Ethiopian, with Shared International Type (SIT) 149, SIT 53, SIT 25, SIT 37, and SIT 21 being the most common SITs (7).

TB has long been a focus of epidemiological studies in Ethiopia. Molecular genotyping approaches are currently being used in such studies to identify mycobacteria species, monitor recent TB transmission, and assess genotype diversity. However, our understanding of TB disease dynamics was limited due to a lack of comprehensive molecular epidemiological data from Ethiopia's peripheral regions, such as the Somali region. Furthermore, because of the inability to distinguish *M. bovis* from *M. tuberculosis* based on routine diagnosis, there is a lack of information regarding the relative contribution of zoonotic TB in pastoral settings, where people live in an environment that allows direct contact with animals or animal products. Therefore, the objective of this study is to investigate the molecular epidemiology of TB in the Somali region of eastern Ethiopia.

## Methodology

### Study area and setting

The study was carried out in health facilities in the Jigjiga city, the capital of the Somali region that is located at 626 km east of Addis Ababa, Ethiopia's capital. The Somali region shares borders with Somaliland, Somalia, Djibouti, and Kenya, as well as local borders with Oromia and Afar regions. More than 83% of the population lives in rural areas, mainly with pastoral or agropastoral livelihood, with livestock serving as the primary source of income. People in the Somali region and those in neighboring countries are ethnically, linguistically, and religiously similar and cross-border movement is common (8, 9). In the Somali region, long-running unrest and insecurity severely impeded the government's capacity to offer basic social services to rural populations (10).

**Figure 1:**
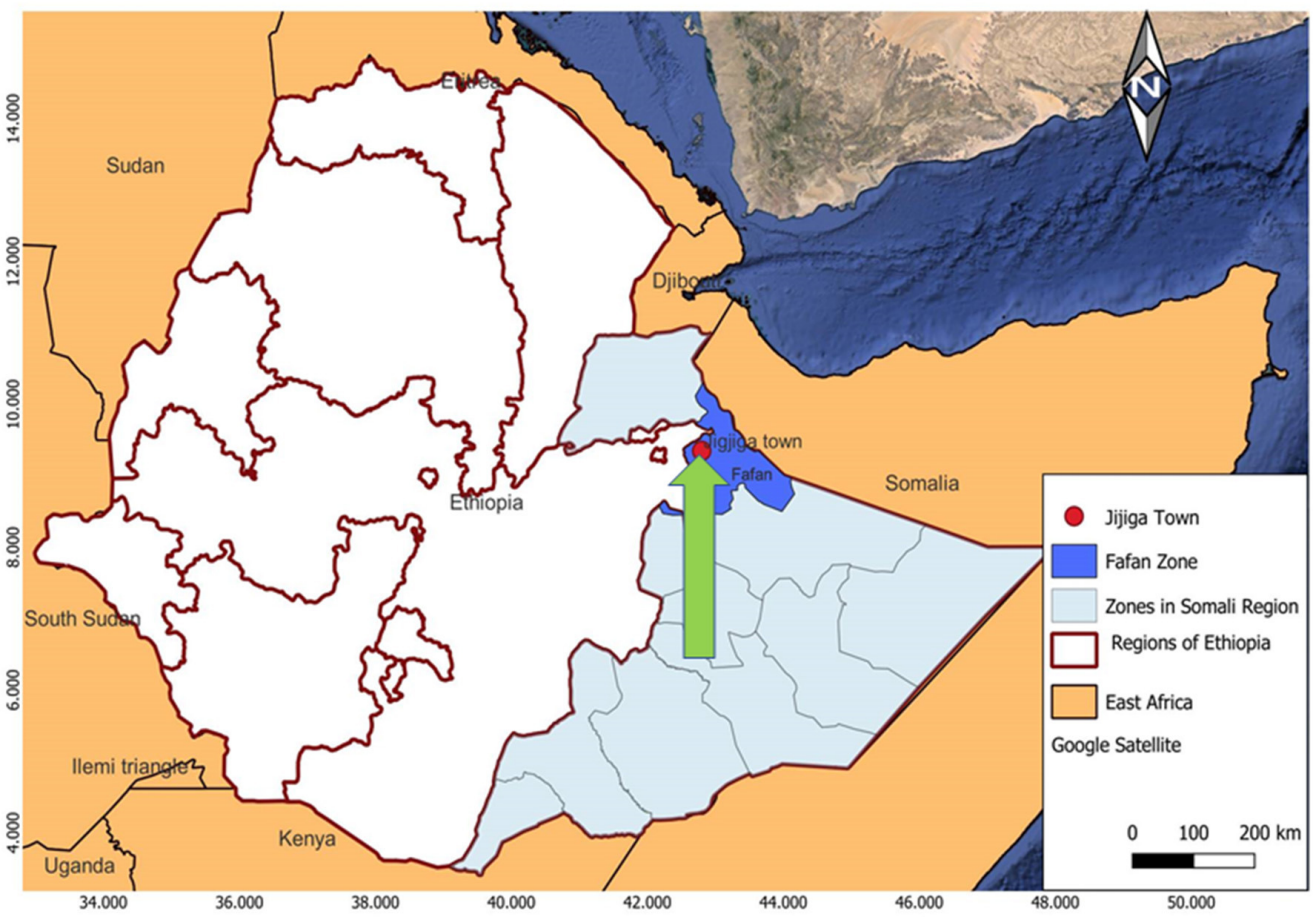
Map of the study area. The arrow indicates the City of Jigjiga that is indicated with a red filled circle. The patients were recruited from three health care provider clinics and hospitals, which are located in the Jigjiga City, namely the Abilelie Health center, Karamara Regional hospital, and Jigjiga University Sheik Hassan Yabare Referral Hospital.

TB patients were recruited from Abilelie Health Center, Karamara Regional Hospital, and Jigjiga University Sheik Hassan Yabare Referral Hospital; all of which are located in Jigjiga City (Figure 1). These hospitals were chosen as they represent the major TB diagnosis and treatment centers in the Region.

### Study design and study subjects

A health institution based, cross-sectional study was conducted on 249 pulmonary and 74 extra-pulmonary TB patients visiting selected health facilities of Jigjiga, Somali region, between October 2018 and December 2019. The study subjects consisted of all consecutive, consenting, bacteriologically confirmed pulmonary TB patients and extra-pulmonary TB patients aged ≥15 years who visited the selected public health institutions. Patients under the age of 15 and those who were unable to produce sputum were excluded from the study.

### Sample collection and mycobacterial culture

Laboratory professionals collected sputum samples from each study participant, as per procedure indicated by WHO (11). If the samples tested positive for acid fast bacilli (AFB) using the Ziehl-Neelsen (ZN) staining method or Xpert MTB/RIF, the remaining portion were kept at −20°C at the sample collection site until transported to Aklilu Lemma Institute of Pathobiology (ALIPB) Addis Ababa University (AAU) TB laboratory. Fine Needle Aspiration (FNA) specimens were collected and evaluated by a pathologist. The first few drops of the aspirates were used for cytological diagnosis, and the rest were preserved at the sample collection site in sterilized phosphate buffer saline in the refrigerator at −20°C. All sputum specimens and FNA were transported in a packed ice box at +4°C to ALIPB TB laboratory, AAU for culture. At each health institute, qualified nurses and laboratory technologists filled out questionnaires to collect patient variables including socio-demographic factors.

The culturing of samples was conducted by following Petroff procedure at ALIPB, AAU (12). The specimens were decontaminated with a final maximum NaOH concentration of up to 2% by centrifuging at 3,000 rpm for 15 min with an equal amount of 4%t NaOH stock solution and sample. The sediment was neutralized with 2NHCl after the supernatant was discarded. The sediment was then inoculated into two conventional Löwenstein–Jensen (LJ) egg slant media, one of which contained 0.6% sodium pyruvate and the other 0.75% glycerol, and cultured for at least 8 weeks with weekly mycobacterial colony observation.

### Molecular identification

DNA was extracted from the mycobacteria grown on LJ media. The bacterial colony from the LJ medium growth was taken and mixed with 200 μl of sterile distilled filtered water. In a water bath, the bacteria and water mixture was heated to 80°C for 1 h (13).

RD9 typing was used to distinguish *M. tuberculosis* species from other members of the MTBC. To determine the presence or absence of RD9 deletion, PCR was performed using three primers: RD9flankF, RD9IntR, and RD9flankR. To identify isolates with deleted RD4, three RD4 primers were used: RD4flankF, RD4IntR, and RD4flankR. The gel was viewed using a Multi-Image Light Cabinet and evaluated based on molecular weight differences; *M. tuberculosis* was recognized by 396-bp intact RD9, whereas other MTBC was recognized by 575-bp deleted RD9. *M. bovis* can be distinguished from other MTBC members by deleted RD4 (446-bp), whereas the other MTBC members have intact RD4 (335-bp) (14).

Spoligotyping was used to characterize all isolates, as described previously by Kamerbeek, following the manufacturer's instructions (Mapmygenome, India) (15). Briefly, DR region was amplified with primers DRa (biotinylated at the 5 “end) and DRb, by Thermal Cycler PCR machine. The amplified product was hybridized to a set of 43 immobilized oligonucleotides, each corresponding to one of the unique spacer DNA sequences within the direct repeat locus. Hybridizing DNA was detected by the enhanced chemiluminescence method and by exposure to X-ray film as specified by the manufacturer. The RD4 and RD9 typing was performed at ALIPB, AAU whereas spoligotyping was performed at Armauer Hansen Research Institute (AHRI), Addis Ababa, Ethiopia.

### Quality control

The sterility of the all culture media was assessed by incubating for 48 h at 37 °C. For RD 9 based PCR and spoligotyping, positive and negative controls were present in each run. H37Rv and BCG strains were used as positive controls, and sterile molecular grade water was used as a negative control. Throughout the study period, a logbook was used to record every laboratory result. The collected data was checked for completeness, accuracy, and clarity before being analyzed and interpreted.

### Identification of spoligotype patterns using SITVIT2 databases

The spoligotyping patterns were changed to binary and octal forms and submitted into an online spoligotype database to identify the SIT number, and the findings were compared to previously designated SIT numbers in the international spoligotyping database (SITVIT2 database) (Pasteur Institute of Guadeloupe), an upgraded version of the previously provided SITVITWEB database (http://www.pasteur-guadeloupe.fr:8081/SITVIT2/) (16). The “TBinsight” database (http://tbinsight.cs.rpi.edu/index.html) was used to designate major lineages. The international database www.mbovis.org was used to compare spoligotype patterns of *M. bovis* isolates.

### Data analysis

All genotyping outputs from the computer analysis were imported into the SPSS 20 computer program and combined with socio-demographic variables. In this study, “clustered” meant two or more isolates with identical spoligotyping patterns, whereas “unique” meant isolates with no common patterns. To demonstrate the socio-demographic variables, descriptive statistics were used. Fisher's exact test was used to analyze the relationship between types of TB and the MTBC clades. The relationships between clustering and associated factors were analyzed using logistic regression analysis. The statistical test was considered significant at *p* < 0.05.

### Ethical clearance

The study was approved by the ALIPB's Institutional Review Board (IRB), AAU Ref No. ALIPB/IRB/002/2017/18.Permission was obtained from the Somali region Health Bureau as well as each study site. Before the samples were collected, the patients were informed about the study and written consent to participate was obtained.

## Results

### Demographic characteristics of the study participants

Among the 323 culture positive TB patients included in this study, 64.4% (208/323) were male, 59.4% (192/323) were urban residents, and 77.1% (249/323) were pulmonary TB patients. Among culture positive extrapulmonary specimens, 95.9% (71/74) were collected from lymph nodes [with cervical lymph node 91.5% (65/71)], 4.0% (3/74) from skin lesion, and 1.3% (1/74) from breast abscess. The median age of the patients was 28 years (range 15–80 years) and 82% (265/323) were in the age group of 15–44 years (Table 1).

**Table 1:** Factors associated with clustering of *M. tuberculosis* complex in Somali, Ethiopia.

		**Clustered**	**Unique**	**AOR (95% CI)**	***p*-value**
Sex	Female	99 (86.1%)	16 (13.9%)	0.90 (0.41–1.95)	0.781
	Male	185 (88.9%)	23 (11.1%)	1	
Residence	Rural	112 (85.5%)	19 (14.5%)	0.73 (0.34–1.60)	0.436
	Urban	172 (89.6%)	20 (10.4%)	1	
Types of TB	Pulmonary TB	224 (90.0%)	25 (10.0%)	2.02 (0.81–4.99)	0.130
	Extra-pulmonary TB	60 (81.1%)	14 (18.9%)	1	
Lineage	Lineage 1	14 (58.3%)	10 (41.7%)	1	
	Lineage 3	88 (95.7%)	4 (4.3%)	15.26 (4.06–57.38)	0.00006*
	Lineage 4	174 (88.8%)	22 (11.2%)	5.41 (2.00–14.60)	0.001*
	Others	8 (72.7%)	3 (27.3%)	2.30 (0.43–12.24)	0.329
Age group	15–24	114 (91.2%)	11 (8.8%)	3.09 (1.13–8.41)	0.03
	25–34	74 (85.1%)	13 (14.9%)	1.47 (0.56–3.84)	0.43
	35–44	49 (92.5%)	4 (7.5%)	2.57 (0.72–9.16)	0.15
	≥45	47 (81%)	11 (19%)	1	

AOR, Adjusted odd ratio; CI, confidence interval. * statistically significant.

### Identification MTBC species and spoligotype patterns

Mycobacterial growth was observed and confirmed by acid fast staining in 323 specimens. The RD9-based PCR analysis showed that 99.7% (322/323, CI: 99.1%-100%) of the isolates had intact RD9 and were classified as *M. tuberculosis*, while 0.3% (1/323, CI: 0–0.9%) had RD9 and RD4 deleted PCR products and were classified as *M. bovis*.

The spoligotype analysis of 323 MTBC isolates based on the SITVIT2 database generated 71 different spoligotype patterns that belonged to 61 shared-types (SITs) with 96.3% (311/323) of the isolates and 10 orphan patterns with 3.7% (12/323) of the isolates. In the Mbovis.org database, the *M. bovis* spoligotype isolated from FNA was SB1942 spoligotype.

The analysis of isolates based on clades/families yields T (lineage 4) account for 41.5% (134/323), CAS (lineage 3) 28.2% (91/323), H (lineage 4) 8.7% (28/323), EAI (lineage 1) 6.8% (22/323), LAM (lineage 4) 6.2% (20/323), and Beijing (lineage 2) 1.9% (6/323) of the all isolates (Table 2). There is no statistically significant association between clade with forms of TB (*p* = 0.062). The major lineages including lineage 1, lineage 2, lineage 3, lineage 4, and lineage 7 were identified in proportions of 7.4, 2.2, 28.2, 60.4, and 0.6%, respectively. Lineage 4 was dominant and comprised of 61.8% (154/249) of pulmonary TB and 56.8% (42/74) of extrapulmonary TB isolates.

**Table 2:** Distribution of spoligotype clades between pulmonary and extrapulmonary TB patients in Somali region, Ethiopia.

	**Pulmonary TB**		**Extrapulmonary TB**		**Total**	
**Clades**	**N (%)**	**95% CI**	**N (%)**	**95% CI**	**N (%)**	**95% CI**
ATYPIC	0	0	1 (1.4)	0–4.1	1 (0.3)	0–0.9
Beijing	3 (1.2)	0–2.8	3 (4.1)	0–9.5	6 (1.9)	0.6–3.4
BOV_1	0	0	1 (1.4)	0–4.1	1 (0.3)	0–0.9
CAS	73 (29.3)	23.7–34.9	18 (24.3)	16.2–35.1	91 (28.2)	23.2–33.1
EAI	16 (6.4)	3.6–9.6	6 (8.1)	2.7–14.9	22 (6.8)	4.3–9.9
Ethiopian	0	0	2 (2.7)	0–6.8	2 (0.6)	0–1.5
Haarlem	23 (9.2)	5.6–13.3	5 (6.8)	1.3–13.8	28 (8.7)	6.2–11.8
LAM	15 (6)	3.2–9.2	5 (6.8)	1.3–13.8	20 (6.2)	3.7–9
MANU	2 (0.8)	0–2	0	0	2 (0.6)	0–1.5
S	1 (0.4)	0–1.2	0	0	1 (0.3)	0–0.9
T	105 (42.2)	36.1–47.8	29 (39.2)	28.4–50	134 (41.5)	36.2–46.7
Ural-1	0		1 (1.4)	0–4.1	1 (0.3)	0–0.9
X	3 (1.2)	0–2.8	0	0	3 (0.9)	0–2.2
Not defined	2 (0.8)	0–2	2 (2.7)	0–6.8	2 (0.6)	0–1.5
Unknown	6 (2.4)	0.8–4.4	1 (1.4)	0–4.1	7 (2.2)	0.6–4
Total	249 (100)		74 (100)		323 (100)	

CI, confidence interval.

Clustered spoligotypes with the size of 2–57 isolates were identified and a majority of the isolates (87.9%; 284/323) (Table 3) were classified under the clustered spoligotypes. The remaining 39 (12.1%) isolates all exhibited unique patterns and thus were singletons (Table 4). The majority of clustered SIT's based on SITVIT classification belonged to the SIT 149 (17.6%), SIT 21 (13.3%), SIT 26 (8.4%), SIT 53 (7.43%), and SIT 52 (5%) which constitute 51.7% of the isolates (Table 3). Evaluation of the association of different factors with clustering demonstrated that lineage 3 and lineage 4, and age group (15–24) were significantly associated with clustering (Table 1).

**Table 3:** Spoligotype patterns of clustered *M. tuberculosis* complex isolates (*n* = 284) TB patients in Somali region, Ethiopia.

**Octal**	**SIT**	**Clade/Subclade**	**Major lineage**	**n (%)**	**Binary code**
777000377760771	149	T3-ETH	Lineage 4	57 (17.6%)	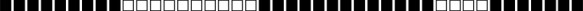
703377400001771	21	CAS1-Kili	Lineage 3	43 (13.3)	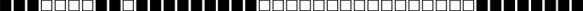
703777740003771	26	CAS1-Delhi	Lineage 3	27 (8.4%)	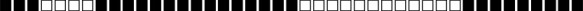
777777777760771	53	T1	Lineage 4	24 (7.4%)	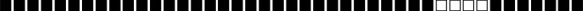
777777777760731	52	T2	Lineage 4	16 (5%)	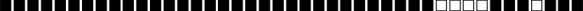
777737777760771	37	T3	Lineage 4	14 (4.3%)	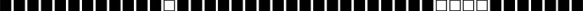
777777775720771	121	H3	Lineage 4	11 (3.4%)	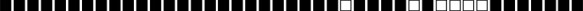
677777607760771	20	LAM1	Lineage 4	10 (3.1%)	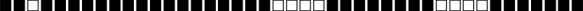
000000000003771	1	Beijing	Lineage 2	6 (1.9)	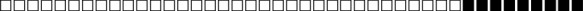
703777740003171	25	CAS1-Delhi	Lineage 3	6 (1.9)	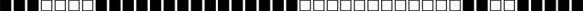
777775777760731	584	T2	Lineage 4	6 (1.9)	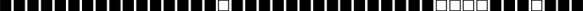
000000007760771	4	Unknown	Lineage 4	5 (1.5%)	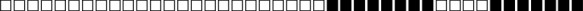
477777277413771	10	EAI8-MDG	Lineage 1	5 (1.5%)	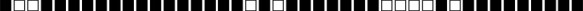
777777774020771	47	H1	Lineage 4	5 (1.5%)	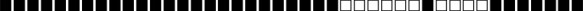
777777777413731	48	EAI1-SOM	Lineage 1	5 (1.5%)	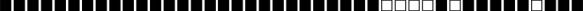
703777700000771	1949	CAS	Lineage 3	5 (1.5%)	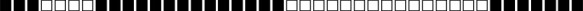
777777607760771	42	LAM9	Lineage 4	4 (1.2%)	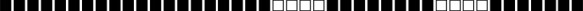
777777777720771	50	H3	Lineage 4	4 (1.2%)	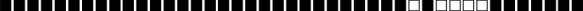
777777777760711	78	T	Lineage 4	4 (1.2%)	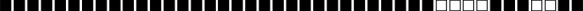
703777740000771	357	CAS1-Delhi	Lineage 3	3 (0.9%)	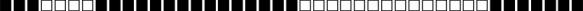
777777377760771	40	T4	Lineage 4	2 (0.6%)	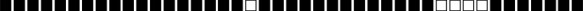
777777777720631	134	H3	Lineage 4	2 (0.6%)	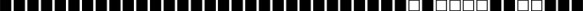
703777740003471	247	CAS1-Delhi	Lineage 3	2 (0.6%)	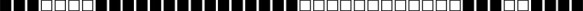
777756777760771	302	X1	Lineage 4	2 (0.6%)	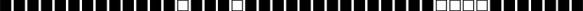
777777757413771	591	EAI6-BGD1	Lineage 1	2 (0.6%)	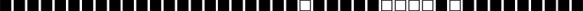
777737747413771	726	EAI6-BGD1	Lineage 1	2 (0.6%)	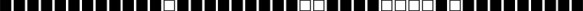
700000007177771	910	Ethiopian	Lineage 7	2 (0.6%)	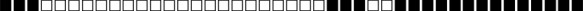
777727777760771	1547	T3	Lineage 4	2 (0.6%)	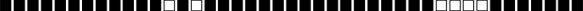
776737777760771	3137	T3	Lineage 4	2 (0.6%)	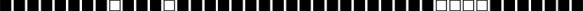
177000377760771	3141	T3-ETH	Lineage 4	2 (0.6%)	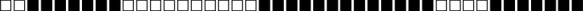

SIT, shared international types.

**Table 4:** Spoligotypes patterns of unique *M. tuberculosis* complex isolates (*n* = 39) from TB patients in Somali region, Ethiopia.

**Octal**	**SIT**	**Sublineage**	**Lineage**	**N (%)**	**Binary code**
477777777413071	11	EAI3-IND	Lineage 1	1 (0.3%)	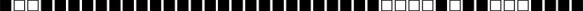
677737607760771	17	LAM2	Lineage 4	1 (0.3%)	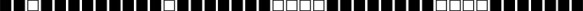
776377777760771	34	S	Lineage 4	1 (0.3%)	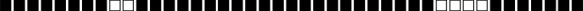
777737777420771	35	Ural-1	Lineage 4	1 (0.3%)	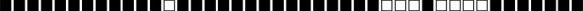
777737777720771	36	H3	Lineage 4	1 (0.3%)	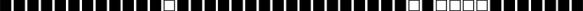
777777747413771	43	EAI6-BGD1	Lineage 1	1 (0.3%)	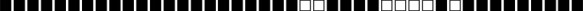
777777777763771	54	Manu2	Lineage 1	1 (0.3%)	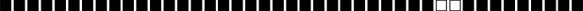
777777607760731	60	LAM4	Lineage 4	1 (0.3%)	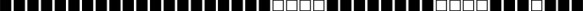
477777377413771	109	EAI8-MDG	Lineage 1	1 (0.3%)	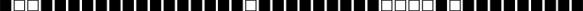
777776777760771	119	X1	Lineage 4	1 (0.3%)	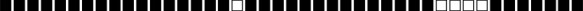
703777700003771	142	CAS1-Delhi	Lineage 3	1 (0.3%)	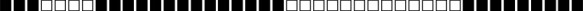
777777777413771	236	EAI5	Lineage 1	1 (0.3%)	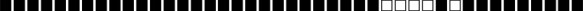
777777777700000	237	Unknown	Lineage 4	1 (0.3%)	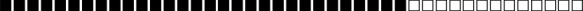
777777704020771	283	H1	Lineage 4	1 (0.3%)	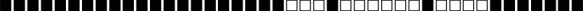
477776077411171	299	EAI3-IND	Lineage 1	1 (0.3%)	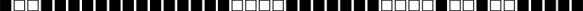
703777740003071	381	CAS1-Delhi	Lineage 3	1 (0.3%)	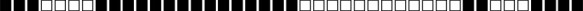
777777777760611	521	T1	Lineage 4	1 (0.3%)	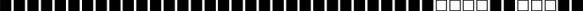
000000007720631	586	H3	Lineage 4	1 (0.3%)	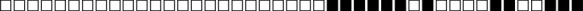
777600007413371	924	EAI5	Lineage 1	1 (0.3%)	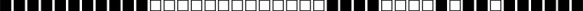
777777377760731	1077	T	Lineage 4	1 (0.3%)	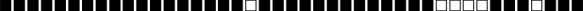
777777777413331	1183	EAI1-SOM	Lineage 1	1 (0.3%)	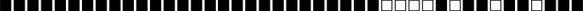
777776607760771	1470	LAM9	Lineage 4	1 (0.3%)	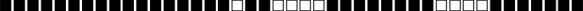
777377777761771	1516	Unknown	Unknown	1 (0.3%)	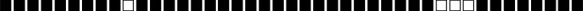
601777606060771	1607	LAM11-ZWE	Lineage 4	1 (0.3%)	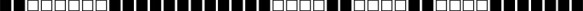
777777777723771	1634	Manu2	Lineage 1	1 (0.3%)	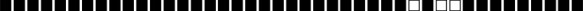
677777607560771	1755	LAM6	Lineage 4	1 (0.3%)	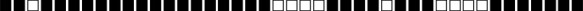
703677740003171	2359	CAS1-Delhi	Lineage 3	1 (0.3%)	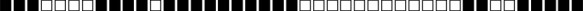
000000000000000	2669	ATYPIC	Lineage 2	1 (0.3%)	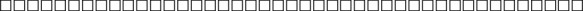
777717774020771	2866	H1	Lineage 4	1 (0.3%)	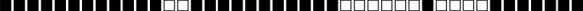
777737377720771	3134	H3	Lineage 4	1 (0.3%)	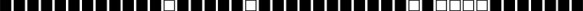
602773777774600	3750	BOV_1	M. bovis	1 (0.3%)	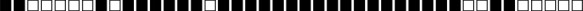
777775745413771	Orphan	EAI6-BGD1	Lineage 1	1 (0.3%)	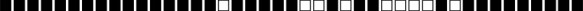
777777177720731	Orphan	H3	Lineage 4	1 (0.3%)	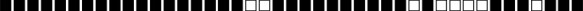
777774007760731	Orphan	LAM4	Lineage 4	1 (0.3%)	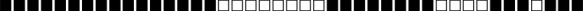
000000020103771	Orphan	Not defined	Unknown	1 (0.3%)	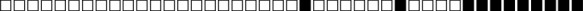
637700003760730	Orphan	Not defined	Lineage 4	1 (0.3%)	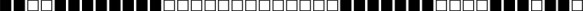
777775477760731	Orphan	Not defined	Lineage 4	1 (0.3%)	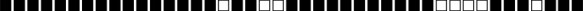
740000000000000	Orphan	Not defined	Unknown	1 (0.3%)	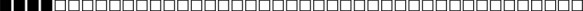
777737767760771	Orphan	T3	Lineage 4	1 (0.3%)	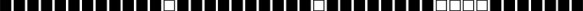

SIT, shared international types.

The anti-TB drugs susceptibility testing was successfully done on 302 isolates using the MGIT960 system. The prevalence of resistance to at least one drug was 11.6%, while the prevalence of MDR-TB was 3.3%. The SIT 149 genotype was the most clustered spoligotype among MDR and significantly associated with both resistance to at least one drug and MDR. The study results have been described previously (17).

## Discussion

Ethiopia has a high TB burden, thus identifying the most frequent MTBC genotypes spreading throughout the country from all types of TB and monitoring the spread of virulent genotypes or zoonotic-transmitted MTBC is crucial (1). To our knowledge this is the first study in the Somali region, investigating MTBC isolates diversity in pulmonary TB and extrapulmonary TB patients.

In this study, MTBC were isolated from pulmonary TB and extrapulmonary TB and the majority of the isolates were *M. tuberculosis*. The role of *M. bovis* was minimal in causing human TB in the Somali region as only one isolate of *M. bovis* was isolated, which is consistent with the observations of the previous studies (18–21). The low rate of human infection by *M. bovis* might be due to the low prevalence of bovine TB in the Somali region. In support of this, Gumi et al. reported a low prevalence of bovine TB among Somali pastoral cattle in southeast Ethiopia (22). Furthermore, human infection requires consumption of contaminated milk from cows with TB mastitis, which affects only about 1% of infected cattle (23, 24). However, Gumi et al. reported three cases of bovine TB among pulmonary TB patients in a study in the southeast Ethiopia pastoral area, suggesting the possibility of aerosol transfer from animal to human or human to human (25). But further study is needed to determine the true burden of zoonotic TB in Ethiopia, as could be a possible source of human TB (21, 25, 26). Thus, in order to end the TB epidemic by 2030, zoonotic TB must be incorporated in prevention efforts (27).

The predominant clades (91.3%) of *M. tuberculosis* isolated in this study belonged to the T, CAS, Haarlem, EAI, and LAM subfamilies. Similar findings were reported from different studies in Ethiopia (18, 28). SIT 149 (T3-ETH) was the most common T subclade identified in our study. Previous studies in Ethiopia also found a high prevalence of SIT 149 (T3-ETH), showing that SIT 149 (T3-ETH) is a dominant subclade in the country (18, 25, 26). As a result, TB epidemic in Ethiopia is mainly driven by the SIT 149 (T3-ETH) spoligotype that spreads across the country. Similar patterns have been found in other African nations where localized genotypes make up a higher proportion of *M. tuberculosis* spoligotypes circulating in those countries (29). The CAS clade was the second most common in our study, and has previously been reported in Ethiopia (30, 31). It is also abundant in Tanzania and Kenya, with SIT 21 (CAS1 KILI) being the most common clade (32).

In the current study, there was no difference in the genotype distribution between pulmonary TB and extrapulmonary TB patients. A previous study in Ethiopia found a similar genetic distribution between the two disease presentations (18). This could suggest that both pulmonary and extrapulmonary TB have similar transmission patterns in the community (23).

Interestingly, the frequency of isolation of the EAI ancestral clade (lineage 1) in this study was higher than the frequencies of its isolation by previously reported studies from the other parts of Ethiopia. Only a few previous studies in Ethiopia reported the EAI clade (25, 33). In the neighboring Somalia, 33.6% of isolates *M. tuberculosis* isolates were reported to be classified under the EAI clade (34), which could suggest that the pastoralists from both countries interact in the border areas on a regular basis as the Somali region of Ethiopia shares a border with Somaliland (Somalia) (9).

Beijing, Ethiopian, ATYPIC, MANU, X, Ural-1, and S genotypes constituted minority group that contributed for a pooled prevalence of 8.4%. The Beijing lineage is widespread in East Asia. In Africa, the Beijing lineage is most common in Southern Africa, although it can also be found sporadically in East Africa (29, 35). The Beijing (SIT 1) clade was detected in our study, and a similar frequency SIT 1 was observed by a study conducted in eastern Ethiopia (33). But the frequency of SIT 1 isolated by the present study was higher than its frequencies of isolation by previous studies from other parts of Ethiopia (7, 21, 28, 36, 37). The relative increase in the frequency of SIT 1 found in this study should considered a serious concern as the STI1 (Beijing) spoligotype is associated with virulence, transmissibility, and drug resistance (6). The prevalence of SIT 910 (lineage 7), which is geographically restricted to Ethiopia, was comparable to the proportion seen in TB patients from South Omo (28), but lower than the proportion found in TB patients from Northwest Ethiopia (38).

The overall clustering rate in this study was high and consistent with the clustering rates reported by the previous studies conducted in Ethiopia (13, 26, 31). A high rate of clustering could suggest active disease transmission in the area. The limited discriminating capacity of spoligotyping, on the other hand, should be noted, and further identification of the isolates using the genotyping method with a better discriminatory power is needed. Multivariable analysis of clustering and major lineages, indicated lineage 3 and lineage 4 were significantly associated with clustering compared with lineage 1 as a reference; clustering was also associated with the younger age group 15–24 compared with age group ≥45 as a reference. The association between the young age group and clustering might be attributed to greater social engagement in the young age group.

Although the low discriminatory power spoligotyping is acknowledged, the genotype data generated by the present study using a large number of *M. tuberculosis* isolates from the Somali region could be considered as useful input to the TB Control Program in the Somali region of Ethiopia, as well as for mapping the population dynamics of MTBC in Ethiopia.

## Conclusion

The MTBC isolates identified from patients with TB in Somali region were highly diverse. The most common lineage types identified in this investigation were lineage 4 and lineage 3, both consisting of clustered spoligotypes, which could suggest the presence of active transmission. In addition, the Beijing spoligotype was isolated from the Somali region of Ethiopia in relatively higher frequency than the frequencies of isolations of the Beijing spoligotype from the other regions of Ethiopia warranting the attention of the TB Control Program of the Somali Region.

## Data availability statement

The original contributions presented in the study are included in the article/Supplementary materials, further inquiries can be directed to the corresponding author.

## Ethics statement

The study was approved by the Institutional Review Board (IRB) of the Aklilu Lemma Institute of Pathobiology, Addis Ababa University Ref No. ALIPB/IRB/002/2017/18. Permission was requested from Ethiopian Somali Regional State Health Bureau and each study sites. The study was explained to the patients, and consent for participation was obtained prior to collecting the specimens. The patients/participants provided their written informed consent to participate in this study.

## Author contributions

GW contributed in designing of the study, data collection, analysis, and drafting of the manuscript. BG supervised the study and edited the manuscript. MG, BM, HS, AW, and WA contributed in the field data collection, culturing of samples, and drug sensitivity testing. RT and LC contributed in edition of the manuscript. GA contributed in conceptualizing and designing of the study, leading and supervision of GW, analysis of and interpretation of the result, and editing of the manuscript. All authors contributed to the manuscript for submission.

## Funding

The research project received small amount of financial support from the American University, USA. In addition, the research project obtained support in kind such as reagents and research supplies from the Ethiopian Public Health Institute (EPHI), Swiss Agency for Development and Cooperation (SDC), Jigjiga University and the Armauer Hansen Research Institute (AHRI) as part of Jigjiga One Health Initiative and Addis Ababa University.

## Conflict of interest

The authors declare that the research was conducted in the absence of any commercial or financial relationships that could be construed as a potential conflict of interest.

## Publisher's note

All claims expressed in this article are solely those of the authors and do not necessarily represent those of their affiliated organizations, or those of the publisher, the editors and the reviewers. Any product that may be evaluated in this article, or claim that may be made by its manufacturer, is not guaranteed or endorsed by the publisher.
